# Linking magmatism with collision in an accretionary orogen

**DOI:** 10.1038/srep25751

**Published:** 2016-05-11

**Authors:** Shan Li, Sun-Lin Chung, Simon A. Wilde, Tao Wang, Wen-Jiao Xiao, Qian-Qian Guo

**Affiliations:** 1 Xinjiang Research Center for Mineral Resources, Xinjiang Institute of Ecology and Geography, Chinese Academy of Sciences, Urumqi 830011, China; 2 Institute of Geology, Chinese Academy of Geological Sciences, Beijing 100037, China; 3 Department of Geosciences, National Taiwan University, Taipei 10617, Taiwan; 4 Xinjiang Key Laboratory of Mineral Resources and Digital Geology, Urumqi 830011, China; 5 Institute of Earth Sciences, Academia Sinica, Taipei 11529, Taiwan; 6 Department of Applied Geology, Curtin University, G.P.O. Box U1987, Perth, Western Australia 6845, Australia; 7 State Key Laboratory of Lithospheric Evolution, Institute of Geology and Geophysics, Chinese Academy of Sciences, Beijing 100029, China; 8 Key Laboratory of Computational Geodynamics, University of Chinese Academy of Sciences, Beijing 100049, China

## Abstract

A compilation of U-Pb age, geochemical and isotopic data for granitoid plutons in the southern Central Asian Orogenic Belt (CAOB), enables evaluation of the interaction between magmatism and orogenesis in the context of Paleo-Asian oceanic closure and continental amalgamation. These constraints, in conjunction with other geological evidence, indicate that following consumption of the ocean, collision-related calc-alkaline granitoid and mafic magmatism occurred from 255 ± 2 Ma to 251 ± 2 Ma along the Solonker-Xar Moron suture zone. The linear or belt distribution of end-Permian magmatism is interpreted to have taken place in a setting of final orogenic contraction and weak crustal thickening, probably as a result of slab break-off. Crustal anatexis slightly post-dated the early phase of collision, producing adakite-like granitoids with some S-type granites during the Early-Middle Triassic (ca. 251–245 Ma). Between 235 and 220 Ma, the local tectonic regime switched from compression to extension, most likely caused by regional lithospheric extension and orogenic collapse. Collision-related magmatism from the southern CAOB is thus a prime example of the minor, yet tell-tale linking of magmatism with orogenic contraction and collision in an archipelago-type accretionary orogen.

Evolution of continental crust is typically marked by a diverse range of magmatism in different tectonic settings^1^. As the final magmatic products of crustal differentiation, granitoids constitute an essential component in the generation of continental crust on Earth^2^. Extraction of granite from the middle-lower crust, and its emplacement at shallower levels, is the principal mechanism by which continental crust has become differentiated^1^. A major setting for granite genesis is in accretionary orogens developed at plate boundaries through subduction processes as a result of transitory coupling across the plate boundary^3^. Post-subduction/accretionary contraction (continent/arc–continent collision) commonly results in disappearance of ocean basins and subsequent shortening and thickening of the crust^2^,^3^, which enables granite extraction, ascent, and emplacement^1^. Thus knowledge of the origin and petrogenesis of granitoids in response to final orogenic contraction in accretionary orogens is essential to understanding material recycling and magmatic processes along convergent margins.

The Central Asian Orogenic Belt (CAOB, Fig. 1a), is widely recognized for its accretionary tectonics and production of massive amounts of juvenile crust in the Phanerozoic, especially in the Paleozoic^4^,^5^,^6^,^7^. It formed by long-lived subduction-accretionary processes from the Mesoproterozoic to the Permian, driven by the evolution and closure of the Paleo-Asian Ocean^4^,^5^,^6^,^7^. Based on distinct geochemical characteristics, the CAOB has been termed an “internal” orogen in contrast to the circum-Pacific “external” orogens^8^. It has also been referred to as a non-collisional orogen, contrasting with the archetypical Alpine-Himalayan collisional orogen^3^. However, all accretionary orogens are ultimately involved in a collisional phase at the end of an orogenic cycle due to ocean closure and termination of subduction, and this may lead to subsequent shortening and thickening of the continental crust^3^. Therefore, collision-related magmatism marks the final episode of a Wilson Cycle, and documents the transition from a convergent plate boundary to intraplate evolution^9^.

The early-middle Paleozoic oceanic subduction/arc magmatism in the southern CAOB has been well documented^10^,^11^. But the time of switching from arc-related magmatism to post-accretionary magmatism is still actively debated. In particular, there is an ongoing controversy with respect to the Permian to Triassic tectonic setting^6^,^12^,^13^. In order to evaluate these changes, we selected the Xilinhot area of Inner Mongolia, China, which consists of, from north to south, the northern accretionary orogen (NAO), the Solonker-Xar Moron (SXM) suture zone and the southern accretionary orogen (SAO)^6^,^10^,^13^ (Fig. 1b). A series of linear or belt granodioritic plutons are present in this area and we selected four of these for SHRIMP U-Pb dating in order to testing they were coeval and perhaps related to the final episode of magmatism associated to closure of the Paleo-Asian Ocean and amalgamation of the southern CAOB. Our study indicates that geochronological, geochemical, and isotopic characteristics of these end-Permian granitoid plutons along the Solonker-Xar Moron suture zone are indeed correlated with the final amalgamation of the CAOB (Figs 1b and 2). Together with other recently-published data from plutonic and volcanic rocks in adjacent areas, we examine the changes in these geochemical parameters in the late Permian-Triassic magmatic rocks, and discuss the interplay between magmatism and orogenesis in the context of closure of the Paleo-Asian Ocean and final continental amalgamation/collision of the South Mongolia Terranes (SMT) with the North China Craton (NCC) to form the largest and most complex Phanerozoic accretionary orogenic belt on Earth.

## Results

### Zircon U-Pb ages

Zircon U-Pb dating by SHRIMP (see Supplementary Dataset 1) yielded weighted mean ^206^Pb/^238^U ages of 255 ± 2 Ma (Beikeli pluton), with one young and discordant ^206^Pb/^238^U age of 241 ± 4 Ma rejected as an outlier; 251 ± 2 Ma (Baiyinwendu pluton) and 252 ± 2 Ma (Sumutai pluton), both devoid of any zircon inheritance; and 253 ± 4 Ma (Salihada pluton), with one 289 ± 4 Ma inherited zircon grain (Figs 2 and 3). Emplacement of these plutons therefore took place at the end-Permian (255–251 Ma).

### Whole rock major and trace elements and Sr-Nd isotopes

The granitoids from the four plutons show a similar range in SiO_2_ (65.3–71.3 wt. %), K_2_O (1.7–2.6 wt. %), Na_2_O (4.5–5.0 wt. %) and CaO (2.4–3.4 wt. %) contents, which indicate calc-alkalic characteristics (Fig. 4a, see Supplementary Dataset 2). The granitoids also have similar contents of Fe_2_O_3_^T^ (1.9–3.4 wt. %) and MgO (0.7–1.7 wt. %), with all samples plotting in the magnesian field (Fig. 4b). The ASI values of all samples range from 1.20 to 1.35, indicating most are weakly peraluminous (Fig. 4c).

All samples exhibit LREE-enriched patterns in the chondrite-normalized rare earth element diagram (Fig. 5a). They show relatively weak REE fractionation ((La/Yb)_N_ = 3.46–9.80)). Most samples show weak negative Eu anomalies (Eu/Eu* = 0.54–0.96) (Fig. 5a), although one sample from the Beikeli pluton (XL922-7.1) and another from the Baiyinwendu pluton (XL921-17) have positive Eu anomalies (Eu/Eu* = 1.26–1.36). The granitoids have low Ni (mostly <10 ppm) and Cr (mostly <35 ppm) contents. In the primitive mantle-normalized spidergram (Fig. 5b), all samples show positive Rb, Th, K and LREE anomalies, and negative Ba, Ta, Nb, P and Ti anomalies (Fig. 5b).

Samples from the four plutons have similar whole-rock Nd-Sr isotopic compositions and record radiogenic Sr_i_ values of 0.7023–0.7037 and positive ε_Nd_(t) values (+2.6 to +3.9) (Fig. 6, see Supplementary Dataset 3), with Neoproterozoic Nd model ages of 0.72–1.10 Ga.

### Zircon Hf-O isotopes

All zircon grains from these samples have positive ε_Hf_(t) values of +8.3 to +14.5 (Figs 6b and 7, see Supplementary Dataset 4) and young two-stage Hf model ages (T_DM2_) of 0.36–0.75 Ga. Samples from the four plutons have variable δ^18^O values of 5.02 to 7.58% (Fig. 8a,b). Of which, the Salihada grantioids record δ^18^O values of 5.85–7.58%, which are higher than those of the other three plutons (Fig. 8a), indicating more recycled supercrustal components were involved.

## Discussion

### Episodes of collision-related magmatic activity

**Figure 1:**
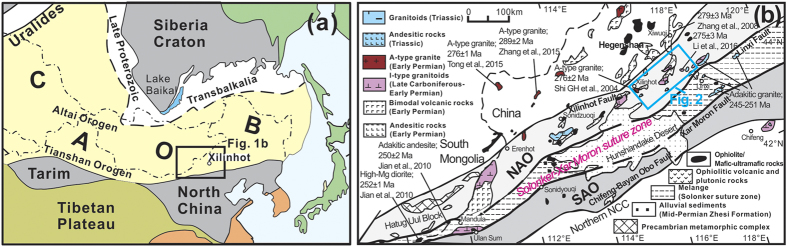
Simplified tectonic map of the Central Asian Orogenic Belt (CAOB) and study region. (**a**) Simplified geological sketch map of the CAOB showing the main tectonic sub-divisions^5^. The location of (**b**) is indicated. (**b**) Simplified tectonic map of the southeastern CAOB showing the main tectonic sub-divisions and the location of Fig. 2 6,12. Light grey zone represents the northern early-middle Paleozoic continental block and the Hutag Uul Block^10^,^12^ or northern accretionary orogen (NAO)^6^, whereas the dark grey zone represents the southern early-middle Paleozoic continental block^10^,^12^ or southern accretionary orogen (SAO)^6^. Published zircon U-Pb ages for early Permian-Triassic magmatic rocks in the region are from refs 12,15, 16, 17,32 and 55. This figure is generated using CorelDRAW X5 (version 15.1.0.588) created by the 2010 Corel Corporation (http://www.corel.com/cn/), and the map will not have a copyright dispute.

**Figure 2:**
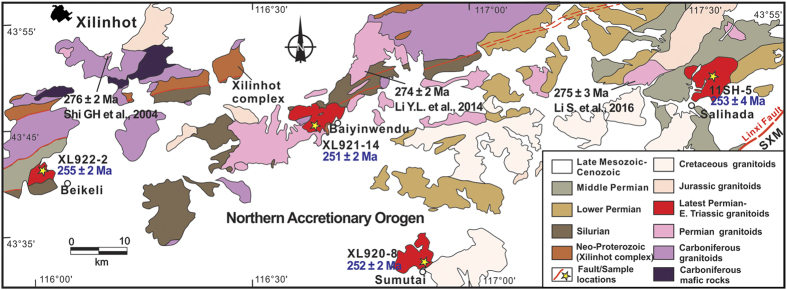
Distribution map of granitoids in the Xilinhot area of Inner Mongolia, China. The NE-trending Carboniferous-Permian granitoid belt and the location of the studied Beikeli, Baiyinwendu, Sumutai and Salihade plutons are shown. Published zircon U-Pb ages for granitoids in the region are from refs 15,31 and 32. This figure is generated using CorelDRAW X5 (version 15.1.0.588) created by the 2010 Corel Corporation (http://www.corel.com/cn/), and the map will not have a copyright dispute.

The southern CAOB has been regarded as a complex tectonic collage of island arcs, accretionary complexes, micro-continental blocks and fragments of oceanic crust that were amalgamated together during the closure of the Paleo-Asian Ocean between the active margin of the South Mongolia Terranes to the north and the northern margin of the North China Craton to the south^6^,^10^,^11^,^12^. Prior to final amalgamation and collision, Late Carboniferous to early Permian (324–272 Ma) arc-signature granitoids and coeval mafic arc complexes with bimodal volcanic rocks formed in the NAO^12^,^14^,^15^,^16^ (Figs 1b and 2). The latest early Permian (277–273 Ma) arc-related granitoids occur as stocks along the southern margin of the NAO that were considered to be derived from an already hybrid andesitic magma in an Andean-type active continental margin^6^,^15^. To the north, an early Permian (292–275 Ma) belt of alkaline granites occurs along the China-Mongolia border^17^ (Fig. 1b). To the south, the Solonker-Xar Moron suture zone is 2500 km long and 50–100 km wide and includes early Permian (299–280) ophiolite complexes, which were stitched together by collision-related igneous rocks (255–248 Ma)^12^, recording final closure of the Paleo-Asian Ocean. A short magmatic hiatus (ca. 270 Ma to 259 Ma) was the result of initial collision^15^. Therefore, four major magmatic episodes along the SXM suture zone can be proposed in response to final amalgamation of the CAOB: subduction controlled (300–273 Ma)^6^,^12^,^15^, slab break-off (255–250 Ma)^12^, intracontinental contraction (251–235 Ma)^13^,^18^, and post-orogenic extension (230–200 Ma)^13^,^14^,^18^.

**Figure 3:**
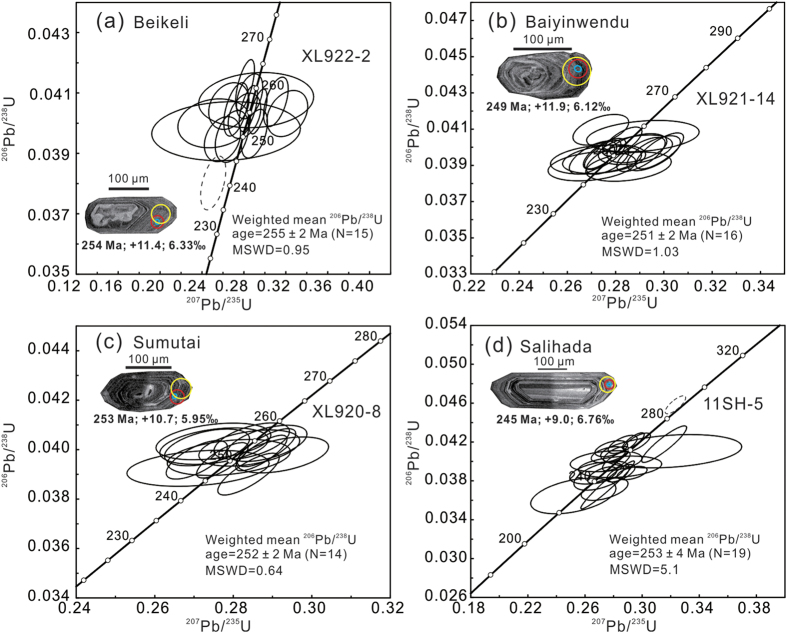
Cathodoluminescence (CL) images of selected zircon grains and U-Pb concordia diagrams. (**a**) Granodiorite (XL922-2) from the Beikeli pluton, (**b**) Granodiorite (XL921-14) from the Baiyinwendu pluton, (**c**) Granodiorite (XL920-8) from the Sumutai pluton, and (**d**) Granodiorite (11SH-5) from the Salihada pluton. Zircon U-Pb ages (red circles), ε_Hf_(t) values (yellow circles) and δ^18^O values (black circles).

The precise zircon U-Pb ages of 255 ± 2 Ma to 251 ± 2 Ma in this study for the four granodioritic plutons (Fig. 3) establish the end-Permian age of granitoids immediately to north of the suture zone. The end-Permian to Triassic was a critical period in the evolution of the CAOB that was punctuated by two major episodes of magmatic activity (255–235 Ma and 230–200 Ma)^18^. Magmatic rocks of the earlier episode are mainly I-type granodiorites (this study) with some S-type granites^19^, adakitic andesite and adakitic granitoids^12^,^20^, sanukitoid-like high-Mg diorite^12^ and E-MORB-like dolerite^12^, which are mainly distributed along the SXM suture zone (Fig. 9). Most show negligible to weak negative Eu anomalies and negative anomalies of Nb and Ta^18^. Magmatic activity associated with the younger episode was sparse, although it has been reported sporadically in the Sonid Zuoqi region^21^, related to Indosinian extension^22^. The rocks are classified as high-K granitoids (222–204 Ma) of A-type affinity with strong negative Eu anomalies. More extensive Late Triassic magmatic activity occurred to the east of the Songliao Basin in Northeast China^14^.

### Geochemical and isotopic constraints on petrogenesis of collision-related magmatic rocks

**Figure 4:**
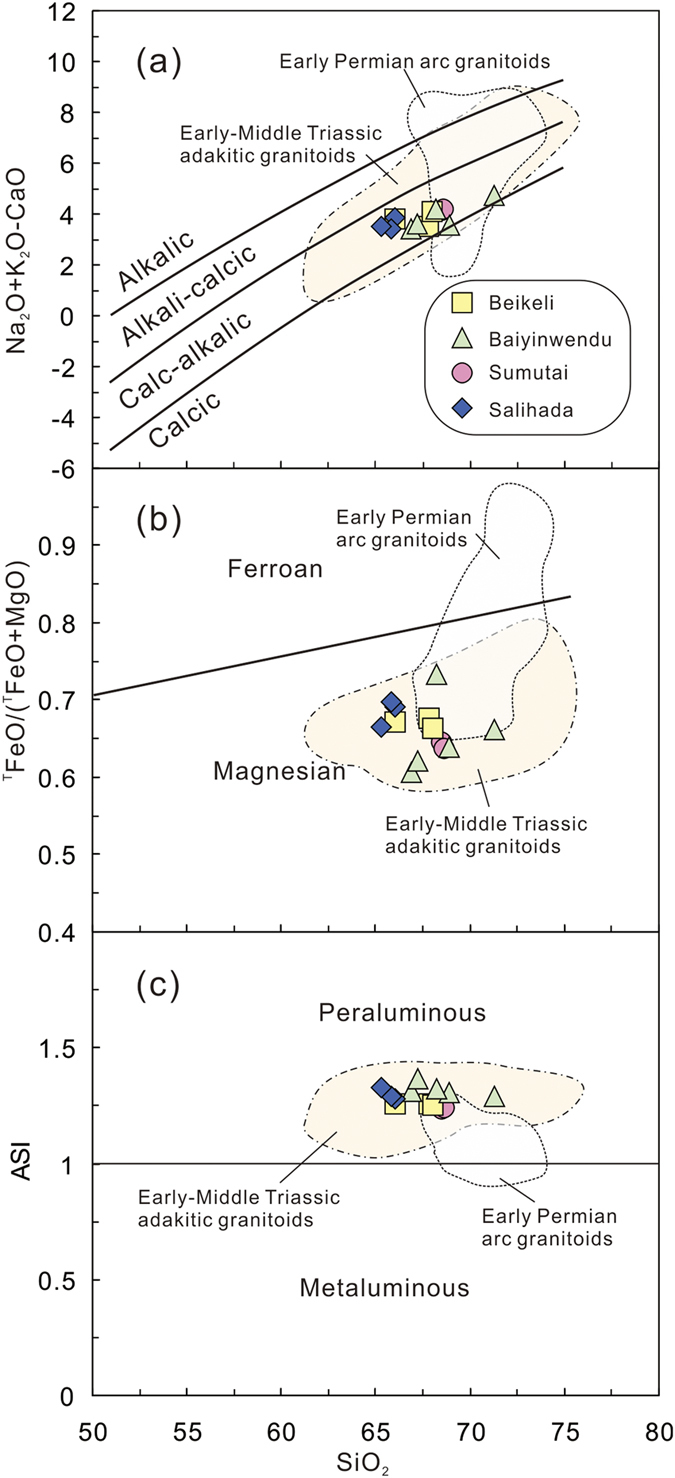
Selected major element diagrams for the Beikeli, Baiyinwendu, Sumutai and Salihada plutons. (**a**) Na_2_O + K_2_O - CaO vs. SiO_2_ diagram^56^, (**b**) ^T^FeO/(^T^FeO + MgO) vs. SiO_2_ diagram^56^ and (**c**) ASI vs. SiO_2_ diagram^56^. ^T^FeO = FeO + 0.9 Fe_2_O_3_, ASI = molecular Al/(Ca − 1.67P + Na + K). Data for the early Permian arc granitoids and Early-Middle Triassic adakitic granitoids are from refs 15,19 and 23.

**Figure 5:**
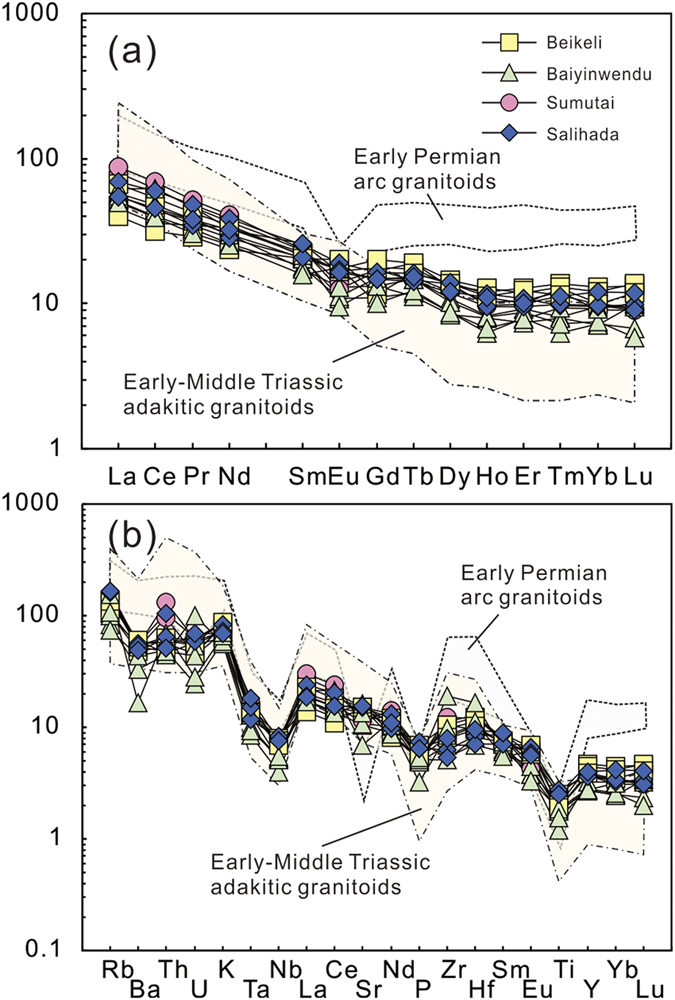
(**a**) Chondrite-normalized rare earth element patterns and (**b**) Primitive mantle-normalized trace element spider diagrams. The values of chondrite and primitive mantle are from Sun and McDonough^57^. Data for the early Permian arc granitoids and Early-Middle Triassic adakitic granitoids are same as in Figure 4.

The end-Permian (255–251 Ma) granitoids record uniform SiO_2_, K_2_O, Na_2_O and CaO contents, have low MgO and Fe_2_O_3_^T^ contents, moderate to weak negative Eu anomalies and low abundances of Ni and Cr, indicating a crustal origin (Fig. 4). They are magnesian granitoids that contain hornblende and have a weak peraluminous nature, characteristic features of I-type granites, although they are generally distinct from the early Permian arc granitoids^15^ (Fig. 4). They also show different REE and trace element patterns from the early Permian arc granitoids, which have higher REE contents and elevated negative Eu and Sr anomalies (Fig. 5). The negative Ta and Nb anomalies of the end-Permian granitoids (Fig. 5b) are a common feature of continental crust produced by geochemical differentiation of arc-derived magmas, and their weakly negative Eu anomalies indicate only minor plagioclase fractionation (Fig. 5a). However, Early-Middle Triassic adakitic granitoids along the suture zone show stronger REE fractionation and lower HREEs^18^,^19^, probably implying residual garnet in the source.

**Figure 6:**
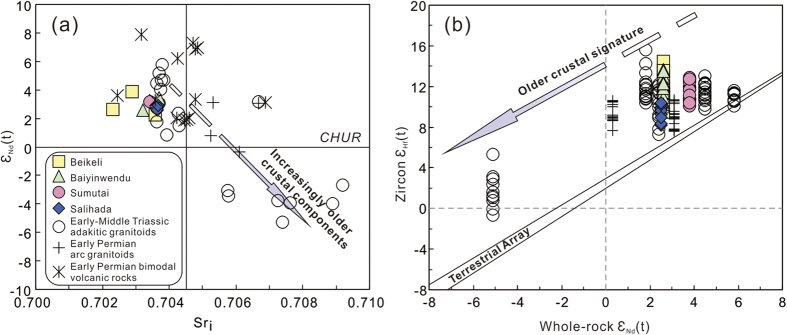
(**a**) ε_Nd_(t) value vs. Sr_i_ value diagram and (**b**) whole-rock ε_Nd_(t) vs. zircon ε_Hf_(t) diagram. Data for the Early-Middle Triassic adakitic granitoids, early Permian arc granitoids and early Permian bimodal volcanic rocks in the area are from refs 15,16, 17, 18,19 and 23. The terrestrial array is from Vervoort and Blichert-Toft^58^.

**Figure 7:**
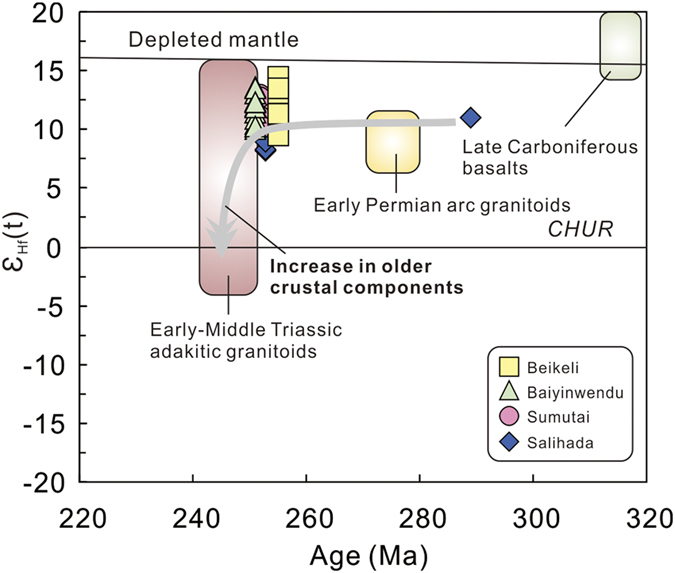
Zircon ε_Hf_(t) vs. U-Pb age diagram. Data for the Early-Middle Triassic adakitic granitoids, early Permian granitoids, and late Carboniferous basalts are from refs 15,18,24 and 59.

The end-Permian granitoids also have low Sr_i_ values (0.7023–0.7037), positive ε_Nd_(t) values (+2.6 to +3.9) (Fig. 6), with Neoproterozoic Nd model ages of 0.72–1.10 Ga, suggesting a predominantly juvenile crustal source. Furthermore, these end-Permian granitoids record positive zircon ε_Hf_(t) values of +8.3 to +14.5 (Figs 6b and 7) and young two-stage Hf model ages (T_DM2_) of 0.36–0.75 Ga, supporting a juvenile crustal source. However, their ε_Nd_(t) values and zircon ε_Hf_(t) values are slightly higher than those of the early Permian arc granitoids in the area^15^, but significantly higher than those of Early-Middle Triassic granitoids^18^,^19^,^23^,^24^ (Figs 6b and 7), indicating greater involvement of young crustal components in their generation. Petrological characteristics and geochemical and isotopic data suggest that those early Permian arc granitoids were mainly derived from juvenile mantle-derived magma mixed with supracrustal materials that had been metasomatically modified by melts/fluids released from the subducting oceanic slab^15^. In addition, the relative enrichment in Sr–Nd–Hf isotopic compositions for the Early-Middle Triassic adakitic granitoids along the SXM suture zone indicate that they likely contained some old continental components, possibly derived from the North China Craton^18^,^19^,^23^,^24^.

**Figure 8:**
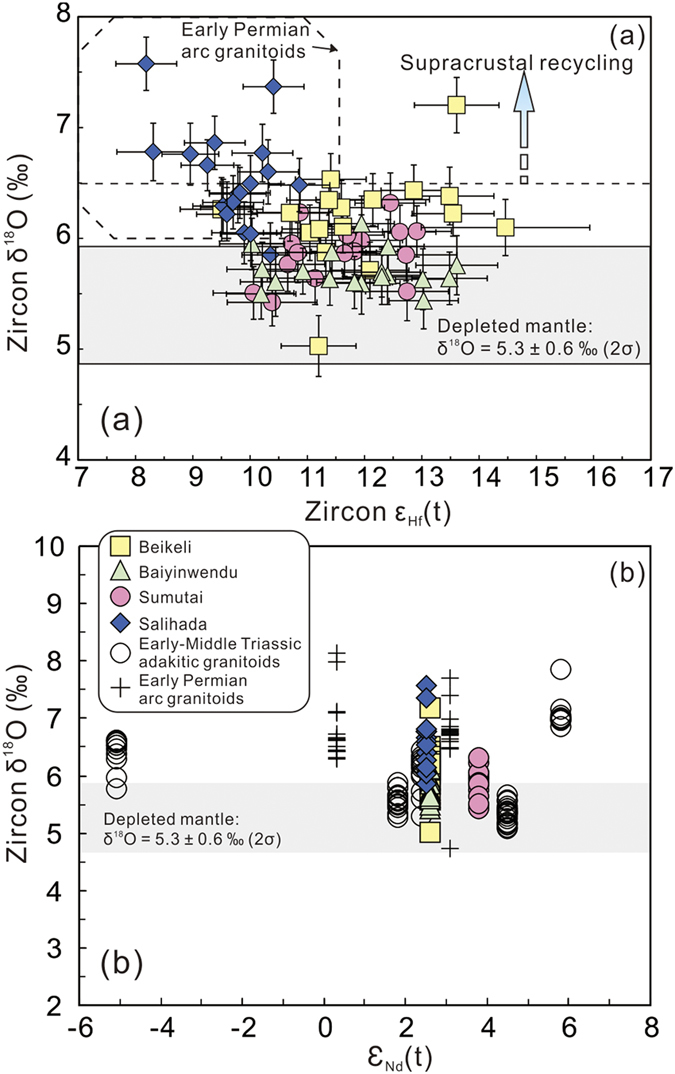
(**a**) zircon δ^18^O vs. Zircon ε_Hf_(t) diagram and (**b**) zircon δ^18^O vs. whole-rock ε_Nd_(t) diagram. The early Permian arc granitoid field is from Li *et al*.^15^.

Three end-Permian plutons show mantle-like to slightly higher δ^18^O values of 5.02 to 6.52%, which are similar to many of those Early-Middle Triassic adakitic granitoids along the SXM suture zone (Fig. 8), suggesting minor involvement of supracrustal materials. However, the end-Permian Salihada grantioids have high δ^18^O values of 5.85–7.58% that are similar to the early Permian arc granitoids (Fig. 8), indicating a greater involvement of supracrustal materials. The isotopic characteristics of these end-Permian granitoids imply a juvenile crustal origin with minor recycled supercrustal materials (sedimentary rocks).

The zircon saturation temperature (T_Zr_) from whole-rock compositions (major element and Zr concentration)^25^ calculated for the end-Permian granitoids yield values of 695–805 °C, with an average of 736 °C. The temperature is distinctly lower than that of the early Permian arc-related granitoids (800–930 °C)^15^, indicating a colder heating mantle by the end of the Permian. However, their low temperature (<800 °C) is similar to that of postcollisional Early-Middle Triassic adakitic granitoids (av. 747 °C) with high Sr/Y ratios (>20) but low Cr (<40 ppm) and MgO, indicating an origin probably from hydrous partial melting of thickened lower crust^26^. The episode of the linear magmatism along the SXM suture zone thus was responsible for orogenic final contraction and collision of the CAOB^18^. As indicated by the whole-rock Nd and zircon Hf isotopic compositions of the end-Permian granitoids (Figs 6 and 7), the participation of juvenile mafic magma in the formation of these granitoids was significant. The detachment of the Paleo-Asian oceanic slab and asthenospheric upwelling through the slab window following the cessation of subduction would therefore trigger partial melting of mafic lower crust to generate these calc-alkaline granitoids. In summary, we suggest that the onset of post-collisional magmatism as a result of slab break-off and asthenospheric upwelling occurred at the end-Permian (See the following discussion).

### A tectono-magmatic scenario of terminal accretion and crustal growth

The four granodioritic plutons examined in this study were emplaced at the end-Permian (between 255–251 Ma) immediately to the north of the SXM suture zone, and coincident with mafic complex (255–248 Ma)^12^ in the Solonker area, and coeval or slightly postdating adakitic granitoids (251–245 Ma)^18^,^19^ along the SXM suture zone, indicating that a narrow linear (~1000 km) magmatic “flare-up” along the suture zone occurred at around 250 ± 5 Ma.

**Figure 9:**
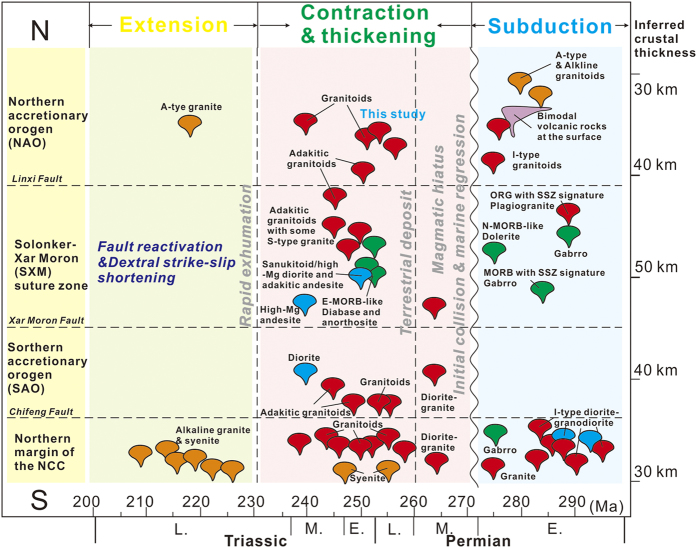
Spatial and temporal distribution of magmatic activity, and relations with major tectonic events during the early Permian to Triassic evolution of the Solonker-Xar Moron suture zone.

Considering that there has been no arc-related magmatism or marine sedimentation along the suture zone since the late Permian^12^,^13^, a subduction-related setting can be ruled out. Furthermore, lithospheric delamination generally results in voluminous magmatism rather than limited linear magmatism, so this too appears unlikely. When the South Mongolia Terranes and the North China Craton collided in the middle-late Permian^6^,^12^, the tensile stresses between the buoyant continental lithosphere and previously-subducted oceanic lithosphere likely led to the separation and detachment of the subducted oceanic slab^27^,^28^,^29^, and slab detachment will result in a narrow, linear zone of magmatism with a limited spatial distribution. Although it remains difficult to explore the geodynamic mechanism responsible for generation of the linear magmatic belt along the SXM suture zone because of the general lack of exposure, the comparable tectonomagmatic events lead us to argue that slab break-off at ca. 255 Ma, soon after a weak arc–continent collision, was a plausible mechanism. Slab break-off records to start with a narrow slab window between the continent and the subducted oceanic slab, resulting in a linear magmatic belt^27^. Such a linear end-Permian magmatic belt, including E-MORB-like dolerite, adakitic andesite, sanukitoid anorthosite, mafic volcanic rocks and I-type granitoids, was distributed along the SXM suture zone (Fig. 9). Jian *et al*.^12^ also proposed that the latest Permian (255–250 Ma) igneous rocks in the Mandula mélange along the SXM suture zone were derived from decompression melting of upwelling asthenosphere from a slab window. The upwelling of asthenosphere during slab break-off can trigger the formation of a variety of magmas, especially tholeiitic basaltic magma^27^,^28^. For example, low-K tholeiitic basalts along the SXM suture zone, that were generated by decompression melting of the asthenosphere, have been identified in the Xilinhot and Linxi areas (ca. 236–252 Ma)^30^, consistent with the slab break-off model. Therefore, a slab break-off model can account for this linear “flare-up” event during the latest Permian to early Triassic along the Solonker-Xar Moron suture zone.

This study focussed in the southern CAOB has wider implications for post-accretionary processes. The end-Permian granitoids along the SXM suture zone show positive ε_Nd_(t) values (+2.6 to +3.9) and positive zircon ε_Hf_(t) values (+8.3 to +14.5), recording significant juvenile crustal input by vertical addition of juvenile magma, with only minor crustal recycling (most δ^18^O values = 5.02 to 6.52%) after closure of the Paleo-Asian Ocean. Our study of the end-Permian granitoids from the southern CAOB thus provides a snapshot of post-accretionary vertical crustal growth in response to final slab break-off.

### Linking magmatism with orogenic processes and tectonic evolution

**Figure 10:**
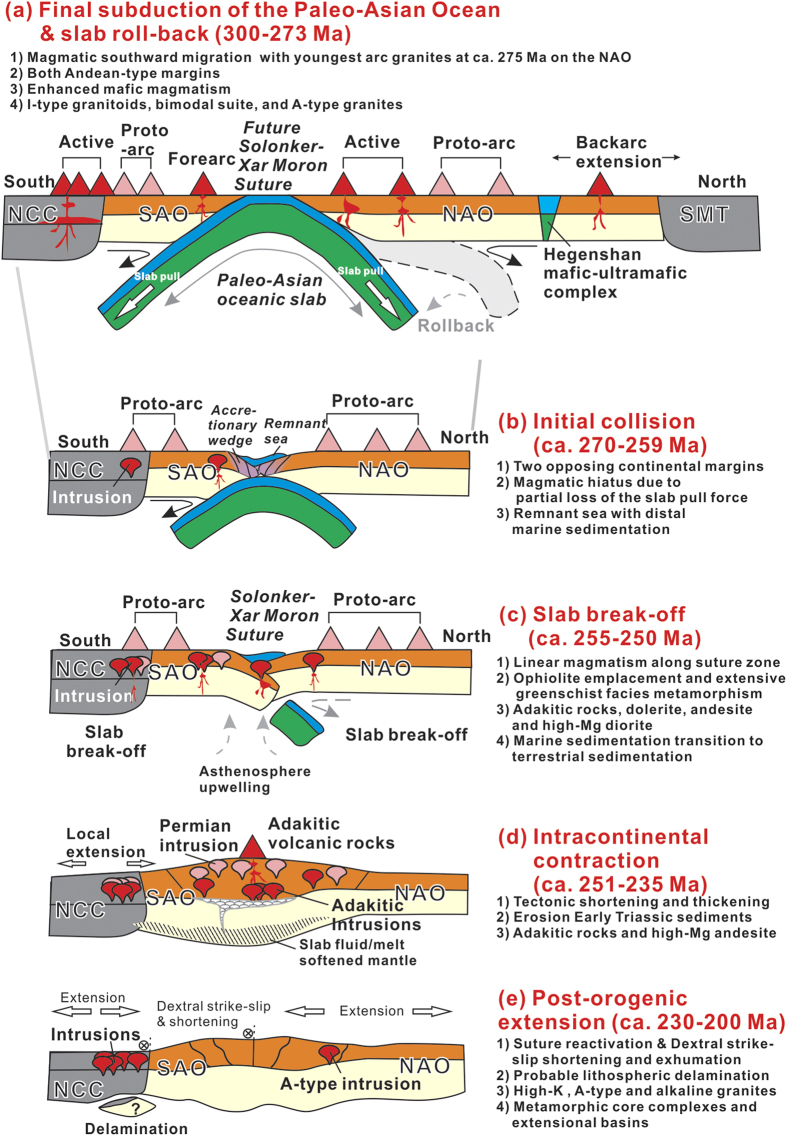
Schematic tectonic diagram showing the final amalgamation of the Central Asian Orogenic Belt and tectono-magmatic evolution along the Solonker-Xar Moron suture zone.

In the early Paleozoic, rocks within the CAOB were generated by the subduction and accretion within the Paleo-Asian Ocean^6^,^7^,^11^, resulting in the formation of the SAO and NAO along the ocean margins, while they were still separated by the Paleo-Asian Ocean^6^,^10^,^12^. During the Carboniferous to early Permian, tectonic activity continued with subduction and arc formation along the Solonker-Xar Moron belt (Fig. 10a)^6^,^12^,^15^. Meanwhile, the outboard migration of arc-related magmas in the NAO was probably responsible for slab retreat and roll-back^15^. Slab roll-back during the early Permian has been interpreted to occur before final closure of the Paleo-Asian Ocean^15^, which induced upper plate (South Mongolia Terranes) extension, causing arc splitting, exhumation of microcontinent slivers (e.g., Xinlin Gol complex) and backarc basins and marginal continental rifting, with calc-alkaline arc^15^,^31^, A-type^32^, alkaline^33^, and bimodal magmatism^16^,^33^ (Figs 9 and 10a). The progressive consolidation of the accreted terranes (mostly early Paleozoic) enabled an Andean-type margin to develop on northern side of the SXM suture zone during the Permian^6^. Coeval with this southward subduction of the Paleo-Asian Ocean beneath the North China Craton a mafic forearc complex formed along the future SXM suture zone^12^, accompanied by Andean-type arc magmatism along the northern margin of the NCC^34^ (Figs 9 and 10a).

Finally, the Paleo–Asian Ocean closed by double-sided subduction in the late Permian, leading to formation of the SXM suture zone^6^,^12^,^15^,^19^,^34^. The available palaeomagnetic data also indicate that the North China Craton and South Mongolia Terranes were very close in the early Permian^35^. Also, a short magmatic hiatus (ca. 270–259 Ma) occurred, during which time a remnant sea with distal marine sedimentation was present along the SXM suture zone^13^,^15^ (Figs 9 and 10b).

This remnant sea likely closed in the Early Triassic, due to contraction between the North China Craton and South Mongolia Terranes, resulting in intermediate P/T greenschist-blueschist facies metamorphism and syn-collisional S-type granites along the SXM suture zone^13^,^19^,^36^ (Figs 9 and 10c). The northern margin of the NCC was also reactivated in the end-Permian to Early Triassic. During this period, the northern margin of the NCC experienced collision-related magmatism, N–S compression, regional exhumation, and uplift of Precambrian crystalline basement, including the formation of E–W-trending south-verging folds and south-verging ductile shear zones^37^. Intense late Permian–Early Triassic shortening along the northern margin of the NCC developed as a result of the collision and contraction of the Central Asian Orogenic Belt^38^,^39^. Therefore, a tectonic switch from early Permian subduction and extension to late Permian contraction along the SXM suture zone was marked by slab break-off at ca. 255–250 Ma (Figs 9 and 10c). The end-Permian to Early Triassic magmatism along the SXM suture zone, likely resulted from partial melting of the mafic lower crust, which was triggered by asthenospheric upwelling through the slab window during the collision-induced break-off of the Paleo-Asian oceanic slab (Figs 9 and 10c). The inferred slab break-off thus marked the end of Paleo-Asian oceanic subduction and termination of the accretionary orogenesis.

Subsequent crustal shortening and thickening, similar in some extent to that of Southern Tibet^40^, is consistent with voluminous Early Triassic sediments being generated from the uplifted orogen in the Linxi area^13^, and formation of lower crust-derived adakite, S-type granite and high-Mg andesite below thickened (>40 km) crust^18^,^19^,^20^ (Figs 9 and 10d). The thickening was focused along the thermally-softened remnant basin, where middle-late Permian sediments were deposited, therefore a shorted-lived (ca. 255–240 Ma) narrow orogen likely formed along what was to become the SXM suture zone, and was squeezed between the older northern accretionary and southern accretionary orogens (Figs 9 and 10d). The stacking of the accretionary wedge above the subduction zone induced the initial slow thickening following slab break-off in the end-Permian, with subsequent faster thickening along the SXM suture zone in the Early Triassic (Fig. 10c,d). Available geological and geophysical evidence suggest that extension of the crust started in the Late Triassic, accompanied by the emplacement of A-type granitic rocks^14^, strike-slip faulting^41^ and formation of metamorphic core complexes^22^. At this time regional lithospheric extension affected the whole of NE China^42^ (Figs 9 and 10e).

## Methods

### Whole-rock geochemical analyses

The samples were crushed after removal of weathered surfaces. The small rock chips were then pulverized into powder using an agate mortar to a grain size of <200 mesh. Whole-rock geochemical analyses were performed at the Analytical Laboratory, Beijing Research Institute of Uranium Geology, China. Major elements were analyzed by X-ray fluorescence spectrometry with a Phillips PW 2404 system. Ferrous iron was determined by the wet chemical titration method. Trace elements (including REE) were determined by inductively coupled plasma-mass spectrometry (ICP-MS). The analytical uncertainties for major element are generally within 1–5%. In-run analytical precision for most trace elements is better than 5%.

### Whole-rock Sr-Nd analyses

The Sr-Nd isotopic compositions were measured by thermal ionization mass spectrometry (TIMS) using a Finnigan MAT-261 mass spectrometer at the Analytical Laboratory, Beijing Research Institute of Uranium Geology, China. The detailed chemical separation and isotopic measurement procedures are described in Wu *et al*.^43^. The ^87^Sr/^86^Sr ratios were normalized to ^86^Sr/^88^Sr = 0.1194, and ^143^Nd/^144^Nd ratios to ^146^Nd/^144^Nd = 0.7219. Total procedural blanks were <300 pg for Sr and <100 pg for Nd, and the estimated analytical uncertainties of ^147^Sm/^144^Nd and ^87^Rb/^86^Sr ratios were <0.5%. The Sr standard solution (NBS 987) was analyzed and yielded ^87^Sr/^86^Sr ratio of 0.710250 ± 14 (2σ), whereas the Nd standard solution (SHINESTU) yielded a ratio of 0.512113 ± 6 (2σ) during data acquisition.

### Zircon U-Pb analyses

Zircon grains were extracted by heavy liquid and magnetic techniques, and further purified by hand-picking under a binocular microscope. They were set in an epoxy mount which was ground and polished to section the zircons in half. Cathodoluminescence (CL) images were taken using a scanning electron microscope at the Beijing SHRIMP Center, Chinese Academy of Geological Sciences, in order to identify any internal structures and to ensure a selection of good analytical sites.

Zircon U-Pb isotope analyses were obtained using the sensitive high resolution ion microprobe (SHRIMP II) at the John de Laeter Centre for Mass Spectrometry, Curtin University, Australia under standard operating conditions (six-scan cycles, 2 nA primary O^2^-beam, mass resolution c.a. 5000), following analytical procedures described by Williams^44^. Inter-element fractionation in the ion emission of zircon was corrected using reference standard TEM2 (416.8 Ma)^45^. Corrections of Pb/U ratios were made by normalization to zircon standard M257 (^206^Pb/^238^Pb = 0.09100, corresponding to an age of 561.3 Ma)^46^. The data were corrected for common lead using the measured ^204^Pb. U-Pb isotope data were calculated and plotted using the SQUID and ISOPLOT software of Ludwig^47^,^48^. The analytical data are presented with 1σ error boxes on the concordia plots and uncertainties in weighted mean ages are quoted at the 95% confidence level (2σ).

### Zircon oxygen isotopic analyses

Zircon oxygen isotopes were measured using the Cameca IMS 1280 at the Centre for Microscopy, Characterisation and Analysis, the University of Western Australia in Perth, and the analytical procedures are similar to those reported by Li *et al*.^49^. The oxygen analysis spots were placed on or adjacent to the SHRIMP pits on the same zircon within a domain of uniform CL. The Cs^+^ primary ion beam was accelerated at 10 kV, with an intensity of 2–3 nA and a spot diameter of about 20 μm. A normal-incidence electron flood gun was used to compensate for sample charging during analysis, with an homogeneous electron density over a 100 μm oval area. Negative secondary ions were extracted with a −10 kV potential. The field aperture was set to 4000 μm, and the transfer-optics magnification was 130. The energy slit width was 30 eV, with a 5 eV gap. The entrance slit width was ca. 110 μm and exit slit width for multi-collector Farady cups (FCs) for ^16^O and ^18^O was 500 μm (MRP = ca. 2200). The intensity of ^16^O^−^ was typically 2 × 109 cps. Oxygen isotopes were measured in multi-collector mode using two off-axis Faraday cups. The Nuclear Magnetic Resonance (NMR) probe was used for magnetic field control stability.

One analysis took ∼4 min consisting of pre-sputtering (∼10 s), automatic beam centering (∼60 s) and integration of oxygen isotopes intensities (20 cycles ×4 s, total 80 s). Uncertainties on individual analyses are reported at the 2σ level and include propagation of uncertainties associated with calculation of instrumental mass fractionation, drift correction, and calculation of δ values relative to Vienna Standard Mean Ocean Water (V-SMOW). The internal precision of a single analysis was generally better than 0.15% for the ^18^O/^16^O ratio. External precision was <0.20% for bracketing standards for all reported analyses. The ^18^O/^16^O ratios are reported in delta notation as δ^18^O values by normalizing to V-SMOW (^18^O/^16^O)_V-SMOW_ = 0.0020052. The internal standard used for correction of mass fractionation was Temora 2 zircon with a δ^18^O value of 8.2 ± 0.01% (1SD)^45^,^46^,^47^,^48^,^49^,^50^.

### Zircon hafnium isotopic analyses

Zircon Hf isotope analyses were carried out using a Newwave UP213 laser-ablation microprobe, attached to a Neptune multi-collector ICP-MS at the Institute of Mineral Resources, Chinese Academy of Geological Sciences, Beijing. Instrumental conditions and data acquisition were as described by Wu *et al*.^51^. The Hf analyses were made on the same spots as the previous oxygen isotope analyses, with a 50 μm spot size. Helium was used as the carrier gas to transport the ablated sample from the laser-ablation cell to the ICP-MS torch and was mixed with argon. In order to correct for isobaric interferences of ^176^Lu and ^176^Yb on ^176^Hf, ^176^Lu/^175^Lu = 0.02658 and ^176^Yb/^173^Yb = 0.796218 ratios were applied^52^. For instrumental mass bias correction, Yb isotope ratios were normalized to ^172^Yb/^173^Yb = 1.35274^52^ and Hf isotope ratios to ^179^Hf/^177^Hf = 0.7325 using an exponential law. The mass bias behavior of Lu was assumed to follow that of Yb, and mass bias correction protocols were as described by Wu *et al*.^43^,^51^. Zircons GJ1 and Plesovice were used as the reference standards during routine analyses, with weighted mean ^176^Hf/^177^Hf ratios of 0.282007 ± 0.000007 (2σ, n = 36) and 0.282476 ± 0.000004 (2σ, n = 27), respectively. These are indistinguishable from the ^176^Hf/^177^Hf ratios of 0.282000 ± 0.000005 (2σ) and 0.282482 ± 0.000008 (2σ), respectively, determined using the solution analysis method by Morel *et al*.^53^ and Sláma *et al*.^54^.

## Additional Information

**How to cite this article**: Li, S. *et al*. Linking magmatism with collision in an accretionary orogen. *Sci. Rep*. **6**, 25751; doi: 10.1038/srep25751 (2016).

## Supplementary Material

Supplementary Information
