# Supplementary material for: Linking magmatism with collision in an accretionary orogen
Source: Sci Rep. 2016 May 11;6:25751. doi: 10.1038/srep25751 (PMC4863176; doi:10.1038/srep25751)
Supplement: Supplementary Information [file srep25751-s1.pdf]

# Linking magmatism with collision in an accretionary orogen

**Shan Li**[1,2,3,4*], **Sun-Lin Chung**[3,5], **Simon A. Wilde**[6], **Tao Wang**[2], **Wen-Jiao Xiao**[1,4,7], **and Qian-Qian Guo**[8]

[1]*Xinjiang Research Center for Mineral Resources, Xinjiang Institute of Ecology and Geography, Chinese Academy of Sciences, Urumqi 830011, China*

[2]*Institute of Geology, Chinese Academy of Geological Sciences, Beijing 100037, China*

[3]*Department of Geosciences, National Taiwan University, Taipei 10617, Taiwan*

[4]*Xinjiang Key Laboratory of Mineral Resources and Digital Geology, Urumqi 830011, China*

[5]*Institute of Earth Sciences, Academia Sinica, Taipei 11529, Taiwan*

[6]*Department of Applied Geology, Curtin University, G.P.O. Box U1987, Perth, Western Australia 6845, Australia*

[7]*State Key Laboratory of Lithospheric Evolution, Institute of Geology and Geophysics, Chinese Academy of Sciences, Beijing 100029, China*

[8]*Key Laboratory of Computational Geodynamics, University of Chinese Academy of Sciences, Beijing 100049, China*

**Corresponding authors:* **Shan Li**
*E-mail address:* lishan428@163.com
*Work telephone number: +86-10-68999834*
*Work fax number: +86-10-68999662*

**Supplementary dataset captions:**

Supplementary dataset 1 (Table S1): SHRIMP U-Pb data for the granitoids in southern Inner Mongolia, China.

Supplementary dataset 2 (Table S2): Geochemical compositions of the end-Permian granitoids from southern Inner Mongolia, China.

Supplementary dataset 3 (Table S3): Sr-Nd isotopic data of the end-Permian granitids from southern Inner Mongolia, China.

Supplementary dataset 4 (Table S4): Zircon Hf-O isotopes of the end-Permian granitoids from southern Inner Mongolia, China.

Table S1 SHRIMP U-Pb data for the granitoids in southern Inner Mongolia, China.

| Analysis spot | Elemental data $^{206}Pb_c$ (%) | $^{206}Pb^*$ | U (ppm) | Th (ppm) | Th/U | Corrected Isotopic Ratios $^{206}Pb^*/^{238}U$ | % | $^{207}Pb^*/^{235}U$ | % | $^{207}Pb^*/^{206}Pb^*$ | % | Age (Ma) $^{207}Pb^*/^{206}Pb^*$ | 1σ | $^{208}Pb^*/^{232}Th$ | 1σ | $^{206}Pb^*/^{238}U$ | 1σ |
|---|---|---|---|---|---|---|---|---|---|---|---|---|---|---|---|---|---|
| Sample XL922-2 from the Beikeli pluton | | | | | | | | | | | | | | | | | |
| 1 | 0.90 | 8.9 | 258 | 107 | 0.43 | 0.0400 | 1.3 | 0.25 | 9.1 | 0.0456 | 9.0 | — | — | 226 | 20 | 253 | 3 |
| 2 | — | 11.2 | 325 | 118 | 0.38 | 0.0403 | 1.2 | 0.32 | 4.0 | 0.0578 | 3.8 | 523 | 83 | 281 | 13 | 255 | 3 |
| 3 | 0.30 | 19.9 | 574 | 188 | 0.34 | 0.0405 | 1.4 | 0.27 | 2.8 | 0.0488 | 2.4 | 141 | 57 | 242 | 8 | 256 | 4 |
| 4 | 0.33 | 15.1 | 434 | 105 | 0.25 | 0.0406 | 1.3 | 0.27 | 4.8 | 0.0475 | 4.6 | 74 | 109 | 232 | 18 | 256 | 3 |
| 5 | 0.08 | 20.5 | 590 | 213 | 0.37 | 0.0405 | 1.2 | 0.28 | 2.0 | 0.0509 | 1.6 | 237 | 37 | 252 | 6 | 256 | 3 |
| 6 | 0.53 | 8.7 | 255 | 96 | 0.39 | 0.0397 | 1.6 | 0.26 | 17.8 | 0.0480 | 17.7 | 99 | 419 | 238 | 45 | 251 | 4 |
| 7 | 0.07 | 6.8 | 195 | 101 | 0.54 | 0.0408 | 1.5 | 0.29 | 12.4 | 0.0519 | 12.3 | 281 | 282 | 247 | 26 | 258 | 4 |
| 8 | 0.48 | 31.4 | 893 | 472 | 0.55 | 0.0409 | 1.2 | 0.29 | 2.9 | 0.0506 | 2.6 | 224 | 61 | 232 | 6 | 258 | 3 |
| 9 | 0.11 | 15.1 | 436 | 235 | 0.56 | 0.0404 | 1.2 | 0.29 | 1.9 | 0.0527 | 1.5 | 317 | 34 | 248 | 5 | 255 | 3 |
| 10 | 0.23 | 16.7 | 483 | 171 | 0.37 | 0.0402 | 1.2 | 0.28 | 3.7 | 0.0506 | 3.4 | 221 | 80 | 250 | 10 | 254 | 3 |
| 11 | 0.73 | 10.5 | 310 | 88 | 0.29 | 0.0395 | 1.2 | 0.26 | 4.4 | 0.0477 | 4.2 | 84 | 100 | 204 | 14 | 250 | 3 |
| 12 | 0.53 | 8.4 | 259 | 81 | 0.32 | 0.0380 | 1.5 | 0.25 | 3.1 | 0.0480 | 2.7 | 99 | 65 | 212 | 8 | 241 | 4 |
| 13 | 0.22 | 18.5 | 522 | 156 | 0.31 | 0.0413 | 1.3 | 0.28 | 2.1 | 0.0494 | 1.6 | 167 | 38 | 238 | 6 | 261 | 3 |
| 14 | — | 16.9 | 483 | 115 | 0.25 | 0.0407 | 1.3 | 0.31 | 2.5 | 0.0553 | 2.1 | 426 | 47 | 293 | 11 | 257 | 3 |
| 15 | 0.48 | 5.0 | 147 | 32 | 0.23 | 0.0396 | 1.4 | 0.27 | 3.9 | 0.0492 | 3.6 | 159 | 85 | 211 | 14 | 250 | 4 |
| 16 | — | 19.7 | 578 | 229 | 0.41 | 0.0397 | 1.3 | 0.29 | 2.8 | 0.0531 | 2.5 | 331 | 56 | 246 | 8 | 251 | 3 |
| Sample XL921-14 from Baiyinwendu pluton | | | | | | | | | | | | | | | | | |
| 2 | — | 7.1 | 205 | 63 | 0.32 | 0.0406 | 1.4 | 0.29 | 5.3 | 0.0520 | 5.1 | 286 | 116 | 254 | 18 | 257 | 4 |
| 3 | 0.25 | 9.9 | 295 | 70 | 0.25 | 0.0389 | 1.4 | 0.27 | 4.4 | 0.0503 | 4.2 | 210 | 98 | 229 | 17 | 246 | 3 |
| 4 | 0.41 | 13.7 | 390 | 112 | 0.30 | 0.0410 | 1.4 | 0.27 | 2.8 | 0.0485 | 2.4 | 123 | 57 | 224 | 8 | 259 | 3 |
| 5 | — | 10.3 | 308 | 70 | 0.23 | 0.0389 | 1.4 | 0.29 | 4.1 | 0.0532 | 3.9 | 336 | 88 | 253 | 18 | 246 | 3 |
| 6 | 0.26 | 13.4 | 397 | 100 | 0.26 | 0.0393 | 1.4 | 0.27 | 4.6 | 0.0501 | 4.4 | 202 | 101 | 227 | 17 | 249 | 3 |
| 7 | 0.06 | 22.8 | 674 | 149 | 0.23 | 0.0393 | 1.3 | 0.28 | 4.1 | 0.0507 | 3.9 | 229 | 90 | 237 | 18 | 249 | 3 |
| 8 | — | 20.1 | 587 | 192 | 0.34 | 0.0399 | 1.3 | 0.29 | 3.5 | 0.0524 | 3.2 | 305 | 74 | 245 | 11 | 252 | 3 |
| 9 | 0.07 | 28.8 | 831 | 347 | 0.43 | 0.0403 | 1.3 | 0.28 | 1.9 | 0.0509 | 1.4 | 236 | 31 | 243 | 5 | 255 | 3 |
| 10 | 0.15 | 18.3 | 538 | 217 | 0.42 | 0.0396 | 1.4 | 0.28 | 2.1 | 0.0504 | 1.6 | 214 | 36 | 237 | 5 | 251 | 4 |
| 11 | 0.02 | 37.8 | 1121 | 259 | 0.24 | 0.0393 | 1.3 | 0.28 | 2.1 | 0.0511 | 1.7 | 245 | 38 | 241 | 7 | 248 | 3 |
| 12 | 0.20 | 19.5 | 577 | 252 | 0.45 | 0.0394 | 1.5 | 0.27 | 2.4 | 0.0498 | 1.9 | 185 | 43 | 241 | 6 | 249 | 4 |

| | | | | | | | | | | | | | | | | | |
|---|---|---|---|---|---|---|---|---|---|---|---|---|---|---|---|---|---|
| 13 | — | 11.2 | 330 | 84 | 0.26 | 0.0397 | 1.6 | 0.30 | 2.5 | 0.0541 | 1.9 | 374 | 42 | 260 | 8 | 251 | 4 |
| 14 | — | 14.6 | 430 | 115 | 0.28 | 0.0396 | 1.6 | 0.29 | 2.3 | 0.0532 | 1.7 | 338 | 38 | 235 | 7 | 250 | 4 |
| 15 | — | 29.5 | 861 | 304 | 0.36 | 0.0399 | 1.3 | 0.29 | 2.8 | 0.0524 | 2.5 | 305 | 57 | 255 | 8 | 253 | 3 |
| 16 | — | 18.2 | 532 | 104 | 0.20 | 0.0398 | 1.7 | 0.30 | 2.2 | 0.0544 | 1.4 | 386 | 32 | 260 | 8 | 252 | 4 |
| 17 | — | 29.9 | 878 | 460 | 0.54 | 0.0396 | 1.3 | 0.27 | 1.8 | 0.0503 | 1.2 | 209 | 29 | 243 | 4 | 250 | 3 |
| Sample XL920-8 from the Sumutai pluton | | | | | | | | | | | | | | | | | |
| 1 | — | 25.7 | 747 | 252 | 0.35 | 0.0401 | 1.3 | 0.29 | 1.7 | 0.0522 | 1.1 | 294 | 26 | 252 | 5 | 253 | 3 |
| 2 | 0.09 | 34.8 | 1021 | 401 | 0.41 | 0.0397 | 1.2 | 0.28 | 3.7 | 0.0510 | 3.5 | 239 | 81 | 248 | 10 | 251 | 3 |
| 3 | 0.30 | 22.0 | 639 | 147 | 0.24 | 0.0401 | 1.2 | 0.28 | 3.3 | 0.0497 | 3.1 | 181 | 72 | 229 | 13 | 254 | 3 |
| 4 | 0.52 | 22.1 | 656 | 462 | 0.73 | 0.0392 | 1.2 | 0.27 | 4.4 | 0.0501 | 4.2 | 199 | 98 | 208 | 7 | 248 | 3 |
| 5 | — | 34.2 | 1000 | 426 | 0.44 | 0.0399 | 1.2 | 0.29 | 1.5 | 0.0522 | 0.9 | 296 | 21 | 252 | 4 | 252 | 3 |
| 6 | — | 27.3 | 790 | 369 | 0.48 | 0.0403 | 1.2 | 0.29 | 2.3 | 0.0520 | 2.0 | 286 | 45 | 249 | 6 | 254 | 3 |
| 7 | 0.42 | 36.8 | 1068 | 900 | 0.87 | 0.0401 | 1.2 | 0.27 | 2.6 | 0.0494 | 2.3 | 169 | 54 | 239 | 4 | 253 | 3 |
| 8 | 0.47 | 48.0 | 1386 | 1093 | 0.81 | 0.0403 | 1.2 | 0.28 | 2.2 | 0.0512 | 1.9 | 249 | 43 | 241 | 4 | 255 | 3 |
| 9 | 0.03 | 32.7 | 962 | 249 | 0.27 | 0.0396 | 1.2 | 0.28 | 1.6 | 0.0508 | 1.0 | 233 | 23 | 243 | 5 | 250 | 3 |
| 10 | 1.39 | 36.3 | 1047 | 624 | 0.62 | 0.0403 | 1.2 | 0.27 | 3.5 | 0.0494 | 3.3 | 169 | 77 | 208 | 6 | 255 | 3 |
| 11 | 0.19 | 26.4 | 767 | 446 | 0.60 | 0.0401 | 1.2 | 0.28 | 3.0 | 0.0514 | 2.7 | 260 | 63 | 237 | 6 | 253 | 3 |
| 12 | — | 22.1 | 645 | 224 | 0.36 | 0.0398 | 1.2 | 0.29 | 2.7 | 0.0533 | 2.4 | 343 | 54 | 261 | 7 | 252 | 3 |
| 13 | 0.14 | 53.5 | 1574 | 698 | 0.46 | 0.0396 | 1.3 | 0.27 | 1.9 | 0.0496 | 1.4 | 176 | 32 | 239 | 4 | 250 | 3 |
| 14 | — | 30.3 | 904 | 353 | 0.40 | 0.0390 | 1.4 | 0.28 | 1.8 | 0.0528 | 1.1 | 319 | 26 | 229 | 4 | 247 | 3 |
| Sample 11SH-5 from the Salihada pluton | | | | | | | | | | | | | | | | | |
| 1 | 0.11 | 23.9 | 694 | 289 | 0.43 | 0.0400 | 1.6 | 0.28 | 2.3 | 0.0511 | 1.6 | 245 | 36 | 247 | 6 | 253 | 4 |
| 2 | — | 17.9 | 539 | 182 | 0.35 | 0.0388 | 1.2 | 0.29 | 2.4 | 0.0548 | 2.1 | 402 | 46 | 256 | 7 | 245 | 3 |
| 3 | 0.30 | 12.7 | 381 | 97 | 0.26 | 0.0388 | 1.9 | 0.27 | 4.6 | 0.0501 | 4.2 | 201 | 98 | 234 | 16 | 245 | 5 |
| 4 | — | 27.6 | 816 | 468 | 0.59 | 0.0394 | 1.8 | 0.29 | 6.8 | 0.0523 | 6.5 | 300 | 148 | 254 | 13 | 249 | 5 |
| 5 | 0.35 | 13.8 | 439 | 117 | 0.27 | 0.0364 | 3.3 | 0.25 | 7.8 | 0.0501 | 7.1 | 200 | 164 | 217 | 24 | 231 | 8 |
| 6 | — | 28.8 | 864 | 391 | 0.47 | 0.0388 | 1.5 | 0.29 | 2.3 | 0.0533 | 1.7 | 342 | 39 | 240 | 6 | 245 | 4 |
| 7 | — | 21.8 | 606 | 245 | 0.42 | 0.0418 | 3.0 | 0.32 | 3.3 | 0.0548 | 1.4 | 402 | 32 | 269 | 9 | 264 | 8 |
| 8 | 0.24 | 15.7 | 474 | 115 | 0.25 | 0.0385 | 2.6 | 0.27 | 4.7 | 0.0511 | 4.0 | 247 | 91 | 236 | 17 | 244 | 6 |
| 9 | 0.02 | 14.6 | 457 | 148 | 0.33 | 0.0371 | 2.0 | 0.27 | 4.0 | 0.0535 | 3.4 | 349 | 77 | 231 | 12 | 235 | 5 |
| 10 | 0.15 | 18.0 | 523 | 176 | 0.35 | 0.0400 | 1.8 | 0.28 | 6.0 | 0.0498 | 5.8 | 186 | 134 | 243 | 18 | 253 | 4 |
| 11 | — | 9.7 | 276 | 114 | 0.43 | 0.0410 | 2.8 | 0.32 | 10.2 | 0.0566 | 9.8 | 476 | 217 | 252 | 29 | 259 | 7 |
| 12 | — | 27.2 | 843 | 232 | 0.28 | 0.0376 | 4.3 | 0.28 | 4.5 | 0.0532 | 1.2 | 339 | 26 | 246 | 12 | 238 | 10 |

| | | | | | | | | | | | | | | | | | |
|---|---|---|---|---|---|---|---|---|---|---|---|---|---|---|---|---|---|
| 13 | 0.03 | 29.2 | 807 | 364 | 0.47 | 0.0421 | 1.3 | 0.29 | 1.7 | 0.0506 | 1.1 | 225 | 25 | 258 | 5 | 266 | 4 |
| 14 | 0.40 | 21.9 | 617 | 252 | 0.42 | 0.0413 | 1.3 | 0.28 | 2.7 | 0.0489 | 2.4 | 142 | 56 | 239 | 7 | 261 | 3 |
| 15 | 0.17 | 20.0 | 554 | 159 | 0.30 | 0.0420 | 1.5 | 0.30 | 2.1 | 0.0512 | 1.5 | 251 | 34 | 251 | 6 | 265 | 4 |
| 16 | 0.45 | 19.3 | 551 | 158 | 0.30 | 0.0407 | 1.3 | 0.28 | 2.9 | 0.0493 | 2.6 | 161 | 60 | 228 | 9 | 257 | 3 |
| 17 | 0.30 | 11.8 | 357 | 102 | 0.30 | 0.0386 | 1.5 | 0.27 | 2.5 | 0.0511 | 2.0 | 247 | 46 | 231 | 7 | 244 | 4 |
| 18 | 0.14 | 14.9 | 425 | 114 | 0.28 | 0.0407 | 1.3 | 0.28 | 2.1 | 0.0502 | 1.7 | 206 | 38 | 243 | 6 | 257 | 3 |
| 19 | — | 61.0 | 1550 | 236 | 0.16 | 0.0458 | 1.3 | 0.33 | 1.4 | 0.0514 | 0.7 | 260 | 15 | 280 | 5 | 289 | 4 |
| 20 | 0.25 | 18.5 | 517 | 197 | 0.39 | 0.0418 | 1.5 | 0.29 | 2.3 | 0.0497 | 1.8 | 183 | 42 | 247 | 6 | 264 | 4 |

Note: *=radiogenic Pb. $^{206}Pb_c$ indicates the common lead portions. Common Pb corrected using measured $^{204}Pb$.

Table S2. Geochemical compositions of the end-Permian granitoids from southern Inner Mongolia, China.

| Pluton | Beikeli pluton | | | Baiyinwendu pluton | | | | | Sumutai pluton | | Salihada pluton | | |
|---|---|---|---|---|---|---|---|---|---|---|---|---|---|
| Sample | XL922-2 | XL922-3 | XL922-7.1 | XL921-7 | XL921-14 | XL921-15 | XL921-16 | XL921-17 | XL920-6 | XL920-8 | 11SH-5 | 11SH-7 | 11SH-8 |
| Major elements (wt.%) | | | | | | | | | | | | | |
| $SiO_2$ | 66.04 | 67.78 | 67.96 | 66.85 | 71.30 | 68.86 | 67.19 | 68.24 | 68.45 | 68.58 | 65.97 | 65.79 | 65.32 |
| $TiO_2$ | 0.54 | 0.43 | 0.41 | 0.39 | 0.26 | 0.38 | 0.41 | 0.34 | 0.41 | 0.42 | 0.59 | 0.60 | 0.55 |
| $Al_2O_3$ | 16.69 | 16.36 | 16.10 | 15.02 | 15.15 | 16.12 | 15.39 | 17.10 | 15.36 | 15.43 | 16.42 | 16.77 | 16.85 |
| $Fe_2O_3$ | 2.25 | 1.70 | 1.50 | 2.45 | 1.10 | 1.80 | 1.65 | 1.40 | 1.60 | 0.50 | 2.20 | 2.50 | 2.45 |
| FeO | 1.11 | 1.02 | 0.95 | 0.14 | 0.68 | 0.62 | 0.57 | 0.71 | 1.00 | 2.16 | 1.17 | 0.87 | 0.78 |
| MnO | 0.06 | 0.05 | 0.05 | 0.05 | 0.04 | 0.05 | 0.05 | 0.05 | 0.05 | 0.05 | 0.05 | 0.05 | 0.06 |
| MgO | 1.59 | 1.25 | 1.20 | 1.67 | 0.87 | 1.33 | 1.32 | 0.74 | 1.37 | 1.39 | 1.45 | 1.42 | 1.59 |
| CaO | 3.37 | 3.35 | 3.11 | 2.75 | 2.35 | 3.02 | 2.60 | 2.97 | 2.96 | 2.94 | 3.20 | 3.41 | 3.21 |
| $Na_2O$ | 4.76 | 4.94 | 4.63 | 4.47 | 4.75 | 4.81 | 4.26 | 5.04 | 4.54 | 4.52 | 4.58 | 4.65 | 4.61 |
| $K_2O$ | 2.43 | 1.90 | 2.60 | 1.70 | 2.31 | 1.77 | 1.97 | 2.11 | 2.57 | 2.63 | 2.48 | 2.19 | 2.10 |
| $P_2O_5$ | 0.15 | 0.11 | 0.12 | 0.11 | 0.07 | 0.11 | 0.11 | 0.12 | 0.11 | 0.11 | 0.14 | 0.16 | 0.14 |
| Los | 0.76 | 0.90 | 1.20 | 4.10 | 0.97 | 0.91 | 4.20 | 1.02 | 1.30 | 1.20 | 1.40 | 1.31 | 1.93 |
| Total | 99.75 | 99.79 | 99.83 | 99.69 | 99.85 | 99.78 | 99.71 | 99.84 | 99.72 | 99.93 | 99.64 | 99.72 | 99.59 |
| Tarce elements (ppm) | | | | | | | | | | | | | |
| La | 10.7 | 15.7 | 9.5 | 12.0 | 13.7 | 13.2 | 14.6 | 11.8 | 18.8 | 20.9 | 16.4 | 13.0 | 12.9 |
| Ce | 21.8 | 32.6 | 19.5 | 24.3 | 29.3 | 26.9 | 37.6 | 23.9 | 37.9 | 42.4 | 36.3 | 27.7 | 27.6 |
| Pr | 3.3 | 4.0 | 2.7 | 3.1 | 3.6 | 3.5 | 4.4 | 2.9 | 4.4 | 4.9 | 4.5 | 3.3 | 3.6 |
| Nd | 15.5 | 16.3 | 11.0 | 12.1 | 14.0 | 14.1 | 15.4 | 11.8 | 17.2 | 19.2 | 18.0 | 13.3 | 15.0 |
| Sm | 3.4 | 3.3 | 3.1 | 2.8 | 3.2 | 2.7 | 3.3 | 2.4 | 3.6 | 3.6 | 3.9 | 3.2 | 3.9 |
| Eu | 1.0 | 1.0 | 1.2 | 0.6 | 0.6 | 0.7 | 0.6 | 1.0 | 0.8 | 0.7 | 1.1 | 1.0 | 1.0 |
| Gd | 3.6 | 4.1 | 2.4 | 2.3 | 3.1 | 2.7 | 2.7 | 2.1 | 3.5 | 3.5 | 3.4 | 3.0 | 3.0 |
| Tb | 0.6 | 0.7 | 0.6 | 0.4 | 0.6 | 0.4 | 0.6 | 0.5 | 0.5 | 0.7 | 0.6 | 0.5 | 0.6 |
| Dy | 3.4 | 3.7 | 3.5 | 2.2 | 2.8 | 2.1 | 2.6 | 2.2 | 3.5 | 3.3 | 3.5 | 3.0 | 3.0 |
| Ho | 0.7 | 0.7 | 0.6 | 0.4 | 0.5 | 0.5 | 0.5 | 0.4 | 0.5 | 0.6 | 0.7 | 0.5 | 0.6 |
| Er | 2.1 | 2.0 | 2.1 | 1.3 | 1.5 | 1.2 | 1.4 | 1.3 | 1.6 | 1.7 | 1.8 | 1.6 | 1.7 |
| Tm | 0.4 | 0.3 | 0.3 | 0.2 | 0.2 | 0.2 | 0.2 | 0.2 | 0.3 | 0.3 | 0.2 | 0.3 | 0.3 |
| Yb | 2.2 | 2.1 | 1.7 | 1.2 | 1.6 | 1.2 | 1.3 | 1.6 | 1.7 | 1.5 | 1.6 | 1.7 | 2.1 |
| Lu | 0.3 | 0.3 | 0.3 | 0.2 | 0.2 | 0.2 | 0.1 | 0.3 | 0.2 | 0.2 | 0.3 | 0.2 | 0.3 |
| Y | 21.5 | 20.1 | 17.9 | 12.2 | 14.6 | 12.6 | 12.8 | 12.4 | 16.9 | 17.0 | 17.2 | 16.4 | 18.2 |
| Sc | 7.0 | 6.6 | 5.0 | 7.2 | 4.8 | 6.0 | 7.0 | 4.5 | 6.7 | 6.8 | 8.8 | 9.0 | 8.5 |
| V | 47.0 | 39.4 | 34.1 | 51.8 | 24.3 | 36.5 | 45.9 | 17.3 | 42.1 | 43.0 | 41.7 | 39.7 | 45.8 |
| Cr | 34.7 | 30.6 | 30.8 | 45.2 | 28.7 | 34.8 | 31.3 | 10.9 | 49.6 | 43.2 | 20.5 | 20.5 | 27.9 |
| Co | 10.3 | 8.3 | 7.2 | 7.5 | 4.9 | 6.8 | 5.1 | 4.0 | 7.0 | 7.1 | 6.6 | 5.2 | 7.6 |
| Ni | 11.6 | 10.5 | 8.5 | 14.0 | 6.5 | 8.4 | 9.4 | 3.5 | 9.1 | 8.4 | 7.7 | 7.4 | 10.9 |
| Ga | 12.6 | 13.7 | 12.2 | 11.8 | 12.3 | 12.9 | 11.9 | 12.7 | 13.7 | 18.1 | 18.2 | 18.6 | 19.7 |
| Rb | 83 | 66 | 85 | 53 | 63 | 47 | 99 | 68 | 90 | 95 | 102 | 104 | 105 |
| Sr | 300 | 323 | 319 | 219 | 225 | 287 | 145 | 342 | 247 | 243 | 322 | 320 | 327 |
| Zr | 118 | 116 | 92 | 212 | 95 | 78 | 58 | 109 | 126 | 139 | 71 | 60 | 88 |
| Nb | 5.7 | 5.5 | 4.9 | 2.8 | 3.8 | 3.8 | 3.7 | 4.0 | 4.8 | 5.1 | 5.7 | 5.9 | 5.4 |
| Cs | 4.6 | 4.9 | 19.2 | 2.1 | 2.2 | 4.0 | 6.8 | 4.5 | 6.4 | 5.4 | 9.0 | 14.0 | 13.0 |
| Ba | 403 | 337 | 417 | 231 | 386 | 303 | 115 | 364 | 357 | 376 | 395 | 374 | 340 |
| Hf | 3.8 | 3.0 | 3.1 | 5.1 | 3.2 | 2.1 | 2.5 | 3.5 | 4.0 | 3.7 | 2.6 | 2.2 | 3.0 |
| Ta | 0.6 | 0.5 | 0.6 | 0.3 | 0.5 | 0.4 | 0.4 | 0.4 | 0.7 | 0.6 | 0.5 | 0.6 | 0.7 |
| Pb | 14.7 | 13.9 | 18.1 | 11.8 | 15.3 | 11.7 | 7.4 | 14.1 | 13.2 | 14.3 | 19.4 | 18.5 | 34.7 |
| Th | 4.8 | 5.4 | 3.9 | 3.8 | 5.2 | 4.2 | 5.8 | 4.1 | 8.1 | 11.1 | 8.8 | 5.5 | 4.4 |
| U | 1.0 | 1.2 | 1.2 | 2.1 | 1.1 | 0.5 | 0.6 | 0.9 | 0.9 | 1.3 | 1.3 | 1.2 | 1.4 |
| ΣREE | 69.0 | 86.7 | 58.5 | 63.1 | 74.8 | 69.8 | 85.2 | 62.3 | 94.7 | 103.6 | 92.3 | 72.3 | 75.6 |
| $(La/Yb)_N$ | 3.46 | 5.44 | 4.05 | 6.94 | 6.10 | 7.76 | 8.18 | 5.39 | 7.84 | 9.80 | 7.31 | 5.62 | 4.49 |
| Eu/*Eu | 0.84 | 0.84 | 1.26 | 0.75 | 0.63 | 0.84 | 0.54 | 1.36 | 0.70 | 0.61 | 0.91 | 0.96 | 0.81 |
| $T_{Zr}$ (ºC) | 743 | 745 | 727 | 805 | 739 | 720 | 704 | 748 | 751 | 759 | 706 | 695 | 728 |

Note: $T_{Zr}$ is the zircon saturation temperature (Watson and Harrison, 1983).

Watson EB & Harrison TM. Zircon saturation revisited: temperature and composition effects in a variety of crustal magma types. *Earth and Planetary Science Letters* **64**, 295-304 (1983).

Table S3. Sr-Nd isotopic data of the end-Permian granitids from southern Inner Mongolia, China.

| Sample | Pluton | Rb (ppm) | Sr (ppm) | $^{87}Rb/^{86}Sr$ | $^{87}Sr/^{86}Sr$ | 2σ | $Sr_i$ | Sm (ppm) | Nd (ppm) | $^{147}Sm/^{144}Nd$ | $^{143}Nd/^{144}Nd$ | 2σ | $f_{Sm/Nd}$ | $\varepsilon_{Nd}(t)$ | $T_{DM}$ (Ga) |
|---|---|---|---|---|---|---|---|---|---|---|---|---|---|---|---|
| XL922-2 | Beikeli | 88 | 291 | 0.875 | 0.705490 | 10 | 0.7023 | 3.31 | 14.00 | 0.1434 | 0.512683 | 7 | -0.27 | 2.6 | 1.01 |
| XL922-3 | Beikeli | 63 | 187 | 0.976 | 0.706432 | 9 | 0.7029 | 3.11 | 14.90 | 0.1263 | 0.512718 | 9 | -0.36 | 3.9 | 0.75 |
| XL922-7.1 | Baiyinwendu | 81 | 303 | 0.773 | 0.706386 | 9 | 0.7036 | 2.62 | 10.70 | 0.1477 | 0.512672 | 14 | -0.25 | 2.3 | 1.10 |
| XL921-14 | Baiyinwendu | 66 | 127 | 1.505 | 0.708589 | 10 | 0.7032 | 2.69 | 13.20 | 0.1232 | 0.512650 | 8 | -0.37 | 2.6 | 0.84 |
| XL921-16 | Baiyinwendu | 99 | 404 | 0.705 | 0.706208 | 11 | 0.7037 | 2.69 | 14.40 | 0.1131 | 0.512678 | 8 | -0.43 | 3.5 | 0.72 |
| XL920-6 | Sumutai | 90 | 248 | 1.044 | 0.707429 | 11 | 0.7037 | 3.27 | 16.60 | 0.1191 | 0.512668 | 11 | -0.39 | 3.1 | 0.78 |
| XL920-8 | Sumutai | 103 | 249 | 1.194 | 0.707699 | 14 | 0.7034 | 3.53 | 18.50 | 0.1154 | 0.512667 | 7 | -0.41 | 3.2 | 0.75 |
| 11SH-5 | Salihada | 93 | 293 | 0.914 | 0.706887 | 11 | 0.7036 | 3.63 | 16.90 | 0.1297 | 0.512660 | 8 | -0.34 | 2.6 | 0.89 |
| 11SH-7 | Salihada | 94 | 291 | 0.936 | 0.706993 | 11 | 0.7036 | 2.94 | 13.20 | 0.1346 | 0.512683 | 8 | -0.32 | 2.9 | 0.90 |

Note: $\varepsilon_{Nd} = ((^{143}Nd/^{144}Nd)_s / (^{143}Nd/^{144}Nd)_{CHUR} - 1) \times 10000$, $f_{Sm/Nd} = (^{147}Sm/^{144}Nd)_s / (^{147}Sm/^{144}Nd)_{CHUR} - 1$, where s = sample, $(^{143}Nd/^{144}Nd)_{CHUR} = 0.512638$, and $(^{147}Sm/^{144}Nd)_{CHUR} = 0.1967$. The model ages ($T_{DM}$) were calculated using a linear isotopic ratio growth equation: $T_{DM} = 1/\lambda \times \ln(1 + ((^{143}Nd/^{144}Nd)s - 0.51315)/((^{147}Sm/^{144}Nd)_s - 0.2137))$.

Table S4. Zircon Hf-O isotopes of the end-Permian granitoids from southern Inner Mongolia, China.

| No. | t (Ma) | $^{176}Yb/^{177}Hf$ | $^{176}Lu/^{177}Hf$ | $^{176}Hf/^{177}Hf$ | $2\sigma_m$ | $\varepsilon_{Hf}(0)$ | $\varepsilon_{Hf}(t)$ | $2\sigma$ | $T_{DM1}$(Hf) (Ga) | $T_{DM2}$(Hf )(Ga) | $\delta^{18}O$ (‰) | $2\sigma$ |
|---|---|---|---|---|---|---|---|---|---|---|---|---|
| Sample XL922-2 from the Beikeli pluton | | | | | | | | | | | | |
| 1 | 255 | 0.038466 | 0.001085 | 0.282962 | 28 | 6.7 | 12.1 | 1.0 | 0.41 | 0.51 | 6.34 | 0.23 |
| 2 | 255 | 0.022575 | 0.000639 | 0.282998 | 22 | 8.0 | 13.5 | 0.8 | 0.36 | 0.42 | 6.37 | 0.26 |
| 3 | 255 | 0.026441 | 0.000791 | 0.282929 | 23 | 5.6 | 11.0 | 0.8 | 0.46 | 0.58 | 6.04 | 0.25 |
| 4 | 255 | 0.033465 | 0.001011 | 0.282935 | 18 | 5.8 | 11.2 | 0.7 | 0.45 | 0.57 | 5.02 | 0.27 |
| 5 | 255 | 0.031143 | 0.001000 | 0.282941 | 17 | 6.0 | 11.4 | 0.6 | 0.44 | 0.55 | 6.52 | 0.23 |
| 6 | 255 | 0.031872 | 0.001017 | 0.282960 | 19 | 6.7 | 12.1 | 0.7 | 0.41 | 0.51 | 5.70 | 0.25 |
| 7 | 255 | 0.027333 | 0.000825 | 0.282945 | 21 | 6.1 | 11.6 | 0.8 | 0.43 | 0.54 | 6.27 | 0.24 |
| 8 | 255 | 0.031468 | 0.000934 | 0.282935 | 18 | 5.8 | 11.2 | 0.7 | 0.45 | 0.57 | 6.07 | 0.24 |
| 9 | 255 | 0.037779 | 0.001213 | 0.282922 | 19 | 5.3 | 10.7 | 0.7 | 0.47 | 0.60 | 6.22 | 0.25 |
| 10 | 255 | 0.026982 | 0.000917 | 0.282946 | 17 | 6.2 | 11.6 | 0.6 | 0.43 | 0.54 | 6.09 | 0.24 |
| 11 | 255 | 0.034632 | 0.001140 | 0.282982 | 16 | 7.4 | 12.9 | 0.6 | 0.38 | 0.46 | 6.42 | 0.23 |
| 12 | 255 | 0.073401 | 0.002162 | 0.282944 | 20 | 6.1 | 11.3 | 0.7 | 0.45 | 0.56 | 5.86 | 0.22 |
| 13 | 255 | 0.026040 | 0.000793 | 0.282939 | 21 | 5.9 | 11.4 | 0.7 | 0.44 | 0.56 | 6.33 | 0.23 |
| 14 | 255 | 0.026163 | 0.000747 | 0.283002 | 21 | 8.1 | 13.6 | 0.7 | 0.35 | 0.41 | 7.19 | 0.25 |
| 15 | 255 | 0.025536 | 0.000664 | 0.282885 | 20 | 4.0 | 9.5 | 0.7 | 0.52 | 0.68 | 6.25 | 0.28 |
| 16 | 255 | 0.030742 | 0.000856 | 0.283026 | 42 | 9.0 | 14.5 | 1.5 | 0.32 | 0.36 | 6.09 | 0.25 |
| 17 | 255 | 0.036203 | 0.000992 | 0.283001 | 20 | 8.1 | 13.5 | 0.7 | 0.36 | 0.42 | 6.21 | 0.20 |
| Sample XL921-14 from the Baiyinwendu pluton | | | | | | | | | | | | |
| 1 | 251 | 0.072844 | 0.002285 | 0.282995 | 17 | 7.9 | 13.0 | 0.6 | 0.38 | 0.45 | 5.43 | 0.25 |
| 2 | 251 | 0.052655 | 0.001666 | 0.282962 | 19 | 6.7 | 11.9 | 0.7 | 0.42 | 0.52 | 5.59 | 0.28 |
| 3 | 251 | 0.050380 | 0.001594 | 0.282974 | 16 | 7.1 | 12.4 | 0.6 | 0.40 | 0.49 | 5.66 | 0.26 |
| 4 | 251 | 0.061487 | 0.001983 | 0.282914 | 17 | 5.0 | 10.2 | 0.6 | 0.49 | 0.63 | 5.49 | 0.23 |
| 5 | 251 | 0.058044 | 0.001908 | 0.282947 | 17 | 6.2 | 11.4 | 0.6 | 0.44 | 0.55 | 5.62 | 0.24 |
| 6 | 251 | 0.024044 | 0.000826 | 0.282988 | 14 | 7.6 | 13.0 | 0.5 | 0.37 | 0.45 | 5.62 | 0.27 |
| 7 | 251 | 0.041648 | 0.001479 | 0.283008 | 20 | 8.3 | 13.6 | 0.7 | 0.35 | 0.41 | 5.75 | 0.27 |
| 8 | 251 | 0.034016 | 0.001185 | 0.282905 | 16 | 4.7 | 10.0 | 0.6 | 0.49 | 0.64 | 5.94 | 0.23 |
| 9 | 251 | 0.056790 | 0.001908 | 0.282976 | 18 | 7.2 | 12.4 | 0.6 | 0.40 | 0.49 | 5.92 | 0.24 |
| 10 | 251 | 0.043648 | 0.001454 | 0.282946 | 22 | 6.2 | 11.4 | 0.8 | 0.44 | 0.55 | 5.86 | 0.25 |
| 11 | 251 | 0.058860 | 0.001872 | 0.282920 | 17 | 5.2 | 10.4 | 0.6 | 0.48 | 0.61 | 5.60 | 0.31 |
| 12 | 251 | 0.036054 | 0.001285 | 0.283003 | 19 | 8.2 | 13.5 | 0.7 | 0.36 | 0.42 | 5.63 | 0.24 |
| 13 | 251 | 0.061362 | 0.001870 | 0.282934 | 19 | 5.7 | 10.9 | 0.7 | 0.46 | 0.58 | 5.70 | 0.21 |
| 14 | 251 | 0.027953 | 0.000922 | 0.282958 | 16 | 6.6 | 11.9 | 0.6 | 0.42 | 0.51 | 6.12 | 0.25 |
| 15 | 251 | 0.050159 | 0.001548 | 0.282958 | 19 | 6.6 | 11.8 | 0.7 | 0.42 | 0.52 | 5.59 | 0.23 |
| 16 | 251 | 0.044827 | 0.001449 | 0.282912 | 17 | 4.9 | 10.2 | 0.6 | 0.49 | 0.63 | 5.71 | 0.24 |
| 17 | 251 | 0.040908 | 0.001258 | 0.282970 | 19 | 7.0 | 12.3 | 0.7 | 0.40 | 0.49 | 5.69 | 0.22 |
| 18 | 251 | 0.034259 | 0.000972 | 0.282968 | 19 | 6.9 | 12.3 | 0.7 | 0.40 | 0.49 | 5.64 | 0.25 |
| Sample XL920-8 from the Sumutai pluton | | | | | | | | | | | | |
| 1 | 252 | 0.042472 | 0.001214 | 0.282959 | 20 | 6.6 | 11.9 | 0.7 | 0.42 | 0.52 | 5.97 | 0.23 |
| 2 | 252 | 0.042823 | 0.001260 | 0.282978 | 18 | 7.3 | 12.6 | 0.6 | 0.39 | 0.47 | 6.05 | 0.24 |
| 3 | 252 | 0.050868 | 0.001571 | 0.282954 | 18 | 6.4 | 11.7 | 0.6 | 0.43 | 0.53 | 6.02 | 0.22 |
| 4 | 252 | 0.040609 | 0.001241 | 0.282925 | 21 | 5.4 | 10.7 | 0.8 | 0.47 | 0.59 | 5.95 | 0.22 |
| 5 | 252 | 0.036779 | 0.001398 | 0.282982 | 25 | 7.4 | 12.7 | 0.9 | 0.39 | 0.47 | 5.84 | 0.23 |
| 6 | 252 | 0.100010 | 0.003179 | 0.282938 | 21 | 5.9 | 10.9 | 0.8 | 0.47 | 0.59 | 6.22 | 0.24 |
| 7 | 252 | 0.049430 | 0.001565 | 0.282988 | 17 | 7.6 | 12.9 | 0.6 | 0.38 | 0.45 | 6.05 | 0.23 |
| 8 | 252 | 0.064765 | 0.001986 | 0.282959 | 21 | 6.6 | 11.8 | 0.8 | 0.43 | 0.52 | 5.87 | 0.21 |
| 9 | 252 | 0.069980 | 0.002141 | 0.282977 | 17 | 7.3 | 12.4 | 0.6 | 0.40 | 0.48 | 6.31 | 0.27 |
| 10 | 252 | 0.054317 | 0.001673 | 0.282925 | 18 | 5.4 | 10.7 | 0.6 | 0.47 | 0.60 | 5.76 | 0.24 |
| 11 | 252 | 0.044875 | 0.001247 | 0.282928 | 20 | 5.5 | 10.8 | 0.7 | 0.46 | 0.59 | 5.86 | 0.27 |
| 12 | 252 | 0.053248 | 0.001429 | 0.282951 | 19 | 6.3 | 11.6 | 0.7 | 0.43 | 0.54 | 5.86 | 0.21 |
| 13 | 252 | 0.035770 | 0.000990 | 0.282904 | 20 | 4.7 | 10.1 | 0.7 | 0.49 | 0.64 | 5.50 | 0.24 |
| 14 | 252 | 0.066336 | 0.001740 | 0.282938 | 24 | 5.9 | 11.1 | 0.8 | 0.45 | 0.57 | 5.63 | 0.24 |

| | | | | | | | | | | | | |
|---|---|---|---|---|---|---|---|---|---|---|---|---|
| 15 | 252 | 0.050363 | 0.001575 | 0.282916 | 22 | 5.1 | 10.4 | 0.8 | 0.48 | 0.62 | 5.42 | 0.21 |
| 16 | 252 | 0.053076 | 0.001403 | 0.282982 | 20 | 7.4 | 12.7 | 0.7 | 0.39 | 0.46 | 5.51 | 0.26 |
| Sample 11SH-5 from the Salihada pluton | | | | | | | | | | | | |
| 1 | 253 | 0.037400 | 0.001200 | 0.282925 | 16 | 5.4 | 10.8 | 0.6 | 0.47 | 0.59 | 6.46 | 0.23 |
| 2 | 253 | 0.030700 | 0.000900 | 0.282888 | 18 | 4.1 | 9.5 | 0.6 | 0.52 | 0.67 | 6.29 | 0.26 |
| 3 | 289 | 0.035800 | 0.001100 | 0.282909 | 18 | 4.8 | 11.0 | 0.6 | 0.49 | 0.61 | 6.56 | 0.26 |
| 4 | 253 | 0.027200 | 0.000900 | 0.282900 | 16 | 4.5 | 9.9 | 0.6 | 0.50 | 0.65 | 6.07 | 0.25 |
| 5 | 253 | 0.008900 | 0.000200 | 0.282851 | 15 | 2.8 | 8.3 | 0.5 | 0.56 | 0.75 | 7.58 | 0.24 |
| 6 | 253 | 0.028400 | 0.000900 | 0.282894 | 16 | 4.3 | 9.7 | 0.6 | 0.51 | 0.66 | 6.25 | 0.28 |
| 7 | 253 | 0.022000 | 0.000700 | 0.282902 | 15 | 4.6 | 10.0 | 0.5 | 0.49 | 0.64 | 6.50 | 0.28 |
| 8 | 253 | 0.024000 | 0.000800 | 0.282884 | 13 | 4.0 | 9.4 | 0.5 | 0.52 | 0.68 | 6.66 | 0.23 |
| 9 | 253 | 0.038600 | 0.001200 | 0.282904 | 16 | 4.7 | 10.0 | 0.6 | 0.50 | 0.64 | 6.05 | 0.28 |
| 10 | 253 | 0.036700 | 0.001200 | 0.282914 | 16 | 5.0 | 10.4 | 0.6 | 0.48 | 0.62 | 7.29 | 0.24 |
| 11 | 253 | 0.023100 | 0.000800 | 0.282899 | 14 | 4.5 | 9.9 | 0.5 | 0.50 | 0.65 | 6.40 | 0.26 |
| 12 | 253 | 0.043500 | 0.001400 | 0.282860 | 18 | 3.1 | 8.4 | 0.6 | 0.56 | 0.74 | 6.78 | 0.26 |
| 13 | 253 | 0.041600 | 0.001300 | 0.282884 | 15 | 4.0 | 9.3 | 0.5 | 0.53 | 0.69 | 6.76 | 0.25 |
| 14 | 253 | 0.020000 | 0.000700 | 0.282889 | 15 | 4.1 | 9.6 | 0.5 | 0.51 | 0.67 | 6.19 | 0.25 |
| 15 | 253 | 0.024200 | 0.000800 | 0.282900 | 15 | 4.5 | 10.0 | 0.5 | 0.50 | 0.64 | 6.41 | 0.23 |
| 16 | 253 | 0.034800 | 0.001100 | 0.282895 | 13 | 4.3 | 9.7 | 0.5 | 0.51 | 0.66 | 6.28 | 0.23 |
| 17 | 253 | 0.017500 | 0.000600 | 0.282913 | 15 | 5.0 | 10.4 | 0.5 | 0.48 | 0.61 | 6.60 | 0.29 |
| 18 | 253 | 0.035400 | 0.001200 | 0.282913 | 15 | 5.0 | 10.3 | 0.5 | 0.48 | 0.62 | 6.77 | 0.26 |
| 19 | 253 | 0.027500 | 0.000900 | 0.282876 | 14 | 3.7 | 9.1 | 0.5 | 0.53 | 0.70 | 6.76 | 0.28 |
| 20 | 253 | 0.030700 | 0.001000 | 0.282916 | 14 | 5.1 | 10.5 | 0.5 | 0.48 | 0.61 | 5.85 | 0.29 |

Note: $\varepsilon_{Hf}(0)=((^{176}Hf/^{177}Hf)/(^{176}Hf/^{177}Hf)_{CHUR,0}-1)\times 10000$, $f_{Lu/Hf}=(^{176}Lu/^{177}Hf)/(^{176}Lu/^{177}Hf)_{CHUR}-1$.
$\varepsilon_{Hf}(t)=((^{176}Hf/^{177}Hf)_s-(^{176}Lu/^{177}Hf)_s\times(e^{\lambda t}-1))/((^{176}Hf/^{177}Hf)_{CHUR,0}-(^{176}Lu/^{177}Hf)_{CHUR}\times(e^{\lambda t}-1))-1)\times 10,000$.
$T_{DM1}(Hf)=1/\lambda\times(1+((^{176}Hf/^{177}Hf)_s-(^{176}Hf/^{177}Hf)_{DM})/((^{176}Lu/^{177}Hf)_s-(^{176}Lu/^{177}Hf)_{DM}))$.
$T_{DM2}(Hf)=T_{DM1}(Hf)-(T_{DM1}(Hf)-t)((f_{CC}-f_S)/(f_{CC}-f_{DM}))$; where, $(^{176}Lu/^{177}Hf)_S$ and $(^{176}Hf/^{177}Hf)_S$ are the measured values of samples; $(^{176}Lu/^{177}Hf)_{CHUR}=0.0332$ and $(^{176}Hf/^{177}Hf)_{CHUR,0}=0.282772$; $(^{176}Lu/^{177}Hf)_{DM}=0.0384$ and $(^{176}Hf/^{177}Hf)_{DM}=0.28325$; $f_{CC}=-0.548$
